# Autologous bone marrow-derived MSCs engineered to express oFVIII-FLAG engraft in adult sheep and produce an effective increase in plasma FVIII levels

**DOI:** 10.3389/fimmu.2022.1070476

**Published:** 2022-12-02

**Authors:** Brady Trevisan, Martin Rodriguez, Hailey Medder, Shannon Lankford, Rebecca Combs, John Owen, Anthony Atala, Christopher D. Porada, Graça Almeida-Porada

**Affiliations:** ^1^ Wake Forest Institute for Regenerative Medicine, Fetal Research and Therapy Program, Wake Forest School of Medicine, Winston-Salem, NC, United States; ^2^ Special Hematology Laboratory, Wake Forest School of Medicine, Winston-Salem, NC, United States

**Keywords:** mesenchymal stroma cell, cell therapy, bone marrow, gene therapy, Hemophilia A, efficacy & safety, FVIII

## Abstract

**Introduction:**

Hemophilia A (HA) is the most common X-linked bleeding disorder, occurring in 1 in 5,000 live male births and affecting >1 million individuals worldwide. Although advances in protein-based HA therapeutics have improved health outcomes, current standard-of-care requires infusion 2-3 times per week for life, and 30% of patients develop inhibitors, significantly increasing morbidity and mortality. There are thus unmet medical needs requiring novel approaches to treat HA.

**Methods:**

We tested, in a highly translational large animal (sheep) model, whether the unique immunological and biological properties of autologous bone marrow (BM)-derived mesenchymal stromal cells (MSCs) could enable them to serve as cellular delivery vehicles to provide long-term expression of FVIII, avoiding the need for frequent infusions.

**Results:**

We show that autologous BM-MSCs can be isolated, transduced with a lentivector to produce high levels of ovine (o)FVIII, extensively expanded, and transplanted into adult animals safely. The transplanted cells engraft in multiple organs, and they stably produce and secrete sufficient quantities of FVIII to yield elevated plasma FVIII levels for at least 15 weeks.

**Discussion:**

These studies thus highlight the promise of cellular-based gene delivery approaches for treating HA.

## Introduction

Bone marrow-derived mesenchymal stromal cells (MSCs) are a multipotent population of cells that can be easily isolated through a non-invasive bone marrow aspiration and can be extensively expanded *in vitro* to generate a large number of cells from a single donor. These cells have been extensively studied in clinical trials for a wide variety of treatments, and these trials have collectively demonstrated that these cells are both safe and well-tolerated (1, 2). MSCs are also relatively immune-inert, and possess a wide range of immunomodulatory effects that cover pathways in both the innate and adaptive branches of the immune system (3). As such, they are uniquely and ideally suited for delivering proteins that have the potential to trigger an immune response upon administration (4). Many of the published studies to-date, both experimental and clinical, have utilized unrelated allogeneic MSCs without evidence of overt immune rejection. However, it has also been shown that administering multiple injections of allogeneic MSCs can result in adverse clinical outcomes (5). Furthermore, even in the absence of any adverse events, transplantation of allogeneic bone marrow derived-MSCs, in adults, has been shown to trigger alloreactivity, suggesting that the immune system is still capable of recognizing and responding to allogeneic MSCs, albeit at a low level. This potential alloreactivity is particularly problematic when using MSCs for cell and gene therapy, as the cells are intended to engraft in large numbers and persist to mediate long-term therapeutic benefit (6). The use of autologous MSCs eliminates these risks/hurdles and would thus greatly facilitate the use of MSCs for gene delivery.

One specific disease for which an immune response to the therapeutic protein is a major clinical challenge is the X-linked bleeding disorder hemophilia A (HA), which can be caused by any one of over 3000 distinct mutations in the F8 gene. Each of these mutations leads to insufficient levels of coagulation Factor VIII (FVIII) protein and/or functional FVIII activity, thereby preventing clotting (7). Although advances in HA therapeutics have improved health outcomes, the current standard of care in many countries still consists of prophylactic infusions of plasma-derived or recombinant FVIII (rFVIII) protein to prevent bleeding events. This treatment is burdensome, as it requires lifelong frequent (2-3 times per week) administration of protein and leads to the development of FVIII-neutralizing IgG antibodies known as FVIII inhibitors in up to 30% of severe HA patients, with patients harboring specific mutations showing higher risk of inhibitor formation (8).

Efficient manufacturing of the multi-domain, highly glycosylated rFVIII protein in non-selected cell lines can be challenging, since the mRNA may not be efficiently expressed, misfolding of the FVIII protein can occur leading to intracellular degradation, and transport of the primary translation product from the endoplasmic reticulum to the Golgi may be inefficient (9, 10).

Thus, the rFVIII is often manufactured in non-human cell lines that have been optimized for high-level protein production, as by increasing the levels of FVIII secretion and concentration in the culture media, the cost of protein production is significantly reduced.

Nonetheless, the glycosylation profile of a protein is determined by the species of the cell that produces the protein, and the use of non-human cell lines in rFVIII production introduces abnormal glycosylation residues that have been implicated in inhibitor induction following administration of recombinant FVIII (11–14). Indeed, the risk of inhibitor formation is thought to be affected by FVIII protein post-translational modifications such as glycosylation and sulfation during the manufacturing process (15).

Using engineered autologous cells as vehicles to deliver FVIII would ensure that the protein being produced and secreted into the circulation has the appropriate native glycosylation profile. As such, the FVIII produced by these cells should exhibit a similar epitope profile to the native FVIII protein, preventing the induction of immunogenic regions on the functional FVIII protein. Indeed, rFVIII products which have been manufactured in human cell lines (16, 17) seem to induce a lower rate of inhibitor formation upon administration (18, 19).

The overall goal of the present study was to use a translational large animal model (sheep) to test the ability of autologous bone marrow (BM)-derived MSCs engineered to produce high levels of ovine FVIII (oFVIII) protein, to engraft and persist in an adult animal, determine the sites and durability of engraftment, and define the duration of expression of the cellularly-encoded oFVIII. We demonstrate that the transplanted cells engrafted in multiple organs, and stably produced and secreted enough FVIII to yield elevated plasma FVIII levels, for at least 15 weeks. These studies thus highlight the promise of using cellular-based gene delivery approaches for providing the missing FVIII protein long-term and as a potential treatment for HA.

## Materials and methods

### Overall study design

The objective of this study was to investigate whether intraperitoneal (IP) administration of autologous BM-derived MSCs transduced with a lentiviral vector to produce high levels of oFVIII-FLAG would be able to engraft and effectively release FVIII into circulation. All animal procedures were performed in accordance with Wake Forest University Health Sciences IACUC guidelines. To determine the animal group size required to achieve sufficient power to detect statistically significant differences between plasma FVIII levels in animals before and after transplant, we used a binary outcome superiority trial (20), set to achieve a 95% chance of detecting, as significant at the 5% level, an increase in plasma FVIII levels as a result of treatment. These calculations confirmed that 3 animals per group were sufficient to achieve the desired statistical power.

MSCs transduced with a lentiviral vector encoding a FLAG-tagged oFVIII transgene were administered *via* ultrasound-guided IP injection (in a single dose of cells producing 60IU/kg/24hr). Blood was collected prior to the first treatment, each week for the next 5 weeks, and then at 7, 10, and 15 weeks after the first treatment. Plasma and mononuclear cells were isolated from the whole peripheral blood at each of these timepoints, and they were then used to determine the plasma FVIII activity and assess the development/presence of an anti-FVIII antibody response within these animals. After the treatments and follow-up timepoints were completed, the animals were euthanized and all major tissues were collected and fixed for histology or stored in AllProtect (Qiagen LLC, Germantown, MD, USA) for RNA and protein analysis.

### Isolation, analysis, and transduction of BM-MSCs

Bone marrow aspirates were collected from healthy adult sheep (n=4) and used to isolate MSCs as previously described (21). Cultured MSCs were analyzed by flow cytometry using directly conjugated antibodies against CD271(Invitrogen, Carlsbad, CA, USA), CD45, CD29, and CD166 (Bio-Rad, Hercules, CA, USA) according to manufacturer’s specifications. Background fluorescence was set using non-specific isotype-matched antibodies and respective fluorochromes. Cells were analyzed using a BD Accuri-C6 and data analyzed using FlowJo software (BD Biosciences San Jose, CA, USA). To determine whether the isolated cells fulfilled the criteria of mesenchymal progenitors, cells were induced to differentiate into adipocytes and osteocytes as previously described (22). In brief, MSCs were plated in MSCGM (Lonza Group AG, Basel, Switzerland) at a density of 5x10^4^/cm^2^ and incubated at 37°C in 5% CO_2_ humidified air. After cells reached 100% confluency, MSCGM was replaced by adipogenic induction media, with induction media changes every 3 days until the presence of cells with vacuoles were detected under microscopy. Cells were rinsed with PBS, fixed with 10% buffered formalin, and stained with Oil Red O. The adipogenic differentiation media consisted of 60 µL of 170 µM Insulin (Sigma, St. Louis, MO, USA), 333 µL of 90 mM IBMX (Sigma), 600 µL of 20 mM Indomethacin (Sigma), 60 µL of 2.5 mM Dexamethasone (Sigma), 9 mL Rabbit serum (Gibco/ThermoFisher, Waltham, MA, USA), and 50 mL M199 (Sigma). After adipogenic induction was achieved, cultures were rinsed gently with sterile PBS (Gibco/ThermoFisher) and fixed with 4% PFA (Polyscience, Niles, IL, USA) for 30-60 minutes at room temperature. Fixed cells were rinsed with deionized (DI) water and incubated with Oil Red O solution (Poly Scientific, Bay Shore, NY, USA) for 20 minutes before rinsing with 60% Isopropanol. Nuclei were then stained using Mayer’s Hematoxylin (Sigma) for 5-10 minutes. Finally, cells were rinsed with DI water and coverslipped using Mounting media (Vector Laboratories, Newark, CA, USA). Osteogenic differentiation was performed by plating 3.1 x 10^3^ MSCs per cm^2^ of tissue culture surface area. Once the MSCs reached 90-100% confluency, MSCGM was replaced with osteogenic induction medium bulletkit (Lonza), and media exchanges were performed every 3-4 days for 2 weeks. At the end of this period, the cells were washed 3 times in PBS, fixed in 95% ethanol for 10 minutes at 4°C, and von Kossa staining was performed to confirm mineralization. The staining solution consisted of 400 µL Naphthol AS-MX Phosphate Alkaline solution (Sigma), 9.6 mL of deionized water, and 2.4 mg of Fast Violet B Salts (Sigma). After the ethanol was removed, the cells were rinsed 3 times with PBS, and cells incubated with the staining solution for 60 minutes at 37°C. To stop the reaction after the 60-minute incubation period, cells were rinsed with DI water, counterstained with hematoxylin (Sigma) for 5 minutes, and rinsed again with DI water. Next, 2.5% silver nitrate (Sigma) solution was added for 30 minutes and cells incubated at 37°C, after which cells were rinsed 3 times with DI water. Finally, cells were allowed to air dry and then stored at 4°C.

Subconfluent cultures of MSCs were transduced twice in QBSF60 (Quality Biologicals, Inc., Gaithersburg, MD, USA) containing 8 µg/mL protamine sulfate (Calbiochem, San Diego, CA, USA) with a VSV-G pseudotyped lentiviral vector pLVX-IRES-Neo encoding oFVIII-FLAG (**Supplemental Figure 1**) at a MOI of 10. The lentiviral vector-containing media was added directly to the cells and incubated for 4 hours at 37°C. After 4 hours, additional lentiviral vector-containing media at a MOI of 10, was added and the flask was returned to the incubator until 24 hours from the initial addition of vector. Cells were washed and placed in MSCGM (Lonza Group AG). These cells were passaged at 70%-80% confluency using TrypLE (Thermo Fisher Scientific) 3 times post-transduction before analysis was performed. The Lenti-X proviral quantitation kit was used as recommended by the manufacturer (Takara Bio USA, Inc., San Jose, CA, USA) to determine vector copy number.

### Preparation of cells for injection

To prepare for transplantation, the transduced MSCs were expanded to the required numbers in gelatin coated CellSTACK chambers (Corning, Corning, NY, USA) in MSCGM. Cells were detached from the flask using TrypLE, washed thoroughly, counted on a Countess machine (Thermo Fisher Scientific), and the required number of cells were resuspended in Plasma-Lyte A (Baxter International, Deerfield, IL, USA). The cells were then drawn into a syringe through a 16-gauge needle, and an extra 1 mL of air was aspirated into the syringe. The needle was then removed from the syringe, and the cell-containing syringe was sealed in a sterile package. During transportation to the transplant facility, the syringe was frequently rotated end-over-end to prevent the cells from clumping.

### IP transplantation of autologous oFVIII-FLAG-MSCs

Sheep recipients were fasted for 24 hours, and prior to the procedure, sheep were anesthetized, the abdominal area was shaved and cleaned with a chlorohexidine scrub (Aspen Veterinary Supplies, Liberty, MO, USA), and ultrasound gel was applied to the area. An echogenic, laser-etched Quincke-tip needle (Havel’s, Cincinnati, OH, USA) was inserted percutaneously into the peritoneal cavity, under continuous ultrasound visualization. Upon successful placement of the needle tip within the peritoneal cavity, the stylet was removed, and the syringe containing the oFVIII-FLAG-MSCs was gently screwed onto the echogenic needle. The entire cell suspension was then slowly administered into the peritoneal cavity (under continuous ultrasound visualization), after which the needle was removed. The animal was then monitored until it had fully regained consciousness. Animals 82, 83, 84, and 89 received a total of 1.27x10^9^, 1.9x10^9^, 5.4x10^9^ and 3.7x10^9^ oFVIII-FLAG-MSCs, respectively, in 5 ml of Plasma-Lyte A (Baxter International).

### Plasma and PBMC isolation

Peripheral blood was collected prior to administering the cellular therapy, as well as each subsequent week for 5 weeks, and at follow up timepoints of weeks 7, 10, and 15 post-cell administration, and plasma and mononuclear cells separated, aliquoted, and frozen.

### Assessment of FVIII pro-coagulant activity

FVIII activity in the plasma of these animals was quantified using aPTT assays as previously described (8) and was performed by the Wake Forest Baptist Medical Center Special Hematology Laboratory in accordance with standard clinical procedures, using a Top 300 CTS clinical coagulometer (Instrumentation Laboratories, Bedford, MA, USA). For each new set of reagents, the value was normalized to a control sample from a previous run to account for possible variation between runs. These values were then normalized to the level of FVIII activity in each animal before the treatment.

### Anti-oFVIII-FLAG IgM and IgG antibody enzyme-linked immunosorbent assay

High-binding plates (Corning) were coated with FVIII/ET3 protein as previously described (23). Sheep plasma samples were serially diluted starting at 1:20 dilution, and antibody binding was detected with anti-ovine IgG : ALP (Bio-Rad Laboratories) or anti-ovine IgM : ALP (Abcam, Cambridge, UK) and *p*-nitrophenyl-phosphate (Bio-Rad Laboratories). The threshold was set as the average of the controls plus 2 standard deviations, which was 0.3 OD for the IgG ELISA and 0.49 OD for the IgM ELISA.

### Anti-FLAG antibody ELISA

FLAG protein in undiluted plasma from these animals was measured with a FLAG-specific ELISA kit, following the manufacturer’s instructions (Cayman Chemical Company, Ann Arbor, MI, USA). FLAG protein in tissues was measured by mincing a small piece of tissue in 500 μL of RIPA buffer (Thermo Fisher Scientific). The resultant tissue suspension was then dissociated with a TissueRuptor II (Qiagen LLC) and spun down at 2500g for 20 minutes to remove any debris. The protein concentration in the supernatant was measured with a NanoDrop 2000 spectrophotometer (Thermo Fisher Scientific) and was then diluted sufficiently to enable assaying 2.5 μg of protein in the same FLAG-specific ELISA kit (Cayman Chemical Company), following the manufacturer’s instructions. 50 μL of protein sample diluted to 0.05 mg/mL (or a known concentration of one of the manufacturer-provided FLAG standards) was added to each well of the plate together with 50 μL of conjugate working solution and antibody. The plate was repeatedly read for absorbance at 650nm until the water-only control well reached an OD of 0.8-1.0, at which time 50 μL of stop solution was added to each well, and the plate was immediately measured on a microplate reader at 450nm.

### Tissue engraftment and tissue protein analysis

Samples from the lung, liver, spleen, mesenteric lymph node, thoracic lymph node, thymus, and ovaries were collected from all animals. Pieces of tissue were placed in fixative to be used for histology or in AllProtect to be stored at -20°C and used for RNA and protein analysis. For histology, the tissue was fixed in neutral buffered formalin for 24 hours before processing and embedding in paraffin. The paraffin-embedded tissue was then sectioned onto glass slides. The slides were baked upright for 60 minutes at 58°C. The tissue was then deparaffinized and rehydrated with an antigen retrieval step at pH:9. The tissue was then permeabilized by adding a few drops of 0.2% Triton-X100 in PBS and incubating for 10 minutes at room temperature. The liquid was then tapped off, and the slides were washed 3 times with 1x TBS. The tissues on the slides were then circled with a hydrophobic pen and incubated for 10 minutes at room temperature with DAKO protein block (Agilent Technologies, Santa Clara, CA, US) to block any non-specific binding. Slides were incubated overnight at 4°C with an antibody specific for the DYKDDDDK (FLAG) tag (Abcam) diluted 1:100 in DAKO reducing antibody diluent (Agilent Technologies). On the following day, the slides were washed 3 times with 1x TBS and incubated for 1 hour in the dark at room temperature with goat anti-rabbit IgG AlexaFluor 594 (Life Technologies, Carlsbad, CA, USA) diluted 1:500 in DAKO antibody diluent. The slides were then washed 3 times with 1x TBST and incubated with TrueBlack^®^ Lipofuscin Autofluorescence Quencher (Biotium, Freemont, CA, USA) for 30 seconds. The slides were washed 3 times with 1x PBS, incubated with DAPI (Thermo Fisher Scientific) diluted 1:1000 in PBS for 5 minutes, washed 3 times with 1x PBS, and mounted with ProLong Gold mounting medium (Thermo Fisher Scientific). An Olympus BX63 microscope (Olympus America, Norfolk, VA) with a 20x objective was used to visualize and capture images of antibody-mediated fluorescence, and a representative image from each tissue was subjected to particle analysis in imageJ (NIH) to quantify the total number of cells and the amount of FLAG protein to determine the quantity of FLAG protein per cell.

### RNA isolation and RT-qPCR analysis

RNA was isolated using a RNeasy Plus Mini Kit after first dissociating the tissue with a TissueRuptor II, and the quality of the RNA was confirmed by 2100 BioAnalyzer with the RNA Nano6000 chip (Agilent Technologies). Samples were only used for RT-qPCR if the RIN was > 7. The RNA was converted into cDNA using an Omniscript RT Kit with Oligo-dT primers (Qiagen LLC). Controls included: a water no template control, animal specific GAPDH controls as internal reference/housekeeping genes, and standard curves of engraftment, including samples of MSC of non-transplanted animals. Normal sheep bone marrow MSCs were used as 0% engraftment. A standard curve of engraftment was created by diluting the transduced cells in normal non-transduced sheep bone marrow MSCs to a final concentration of 0.1%, 1%, 5%, 10%, and 15% (by cell count). A master mix of 10 μL TB Green Advantage and 0.4 μL ROX LMP (Takara Bio USA) was prepared for each well of the PCR plate. Primers to either oFVIII-FLAG or sheep GAPDH were added to a final concentration of 350 nM or 200 nM, respectively, and UltraPure Distilled Water (Thermo Fisher Scientific) was added to enable 18 μL of one of these two master mixes to be added to each well followed by 2 μL of sample at a 5 ng/μL concentration. The plate was run on a QuantStudio 6 Flex Real-Time PCR System (Thermo Fisher Scientific). The reaction conditions consisted of a hold stage for 10 seconds at 95˚C, followed by 40 cycles consisting of 4 seconds at 95˚C and 25 seconds at 60˚C. After 40 cycles were completed, a melt curve stage was run at 95˚C for 15 seconds followed by 60˚C for 1 minute and 95˚C for 15 seconds. The CT values for the amplification curves obtained with the FLAG-specific primers for each sample were normalized to those obtained with the primers to sheep GAPDH to calculate a ΔCT for each tissue and each standard. The standard curve was then used to create a regression line, and an equation to calculate percent engraftment from ΔCT. This equation was then used to determine the percent engraftment for each sample.

### Technical and biological replicates

Technical replicates consisted of assays performed on MSCs isolated from at least 2 different BM harvests from each animal, assays performed on cells expanded at different times, and/or assays performed three times for each sample, depending on the experiments. Biological replicates refer to the number of different animals analyzed in each experiment.

### Statistical analyses

Experimental results are presented as the mean plus/minus the standard error of the mean (SEM). All statistical analyses were performed using the R coding language in RStudio (RStudio, PBC, Boston, MA). One-way ANOVA was employed for multiple comparisons. A p value <0.05 was considered statistically significant.

## Results

### Characterization of sheep bone marrow mesenchymal stromal cells

**Figure 1:**
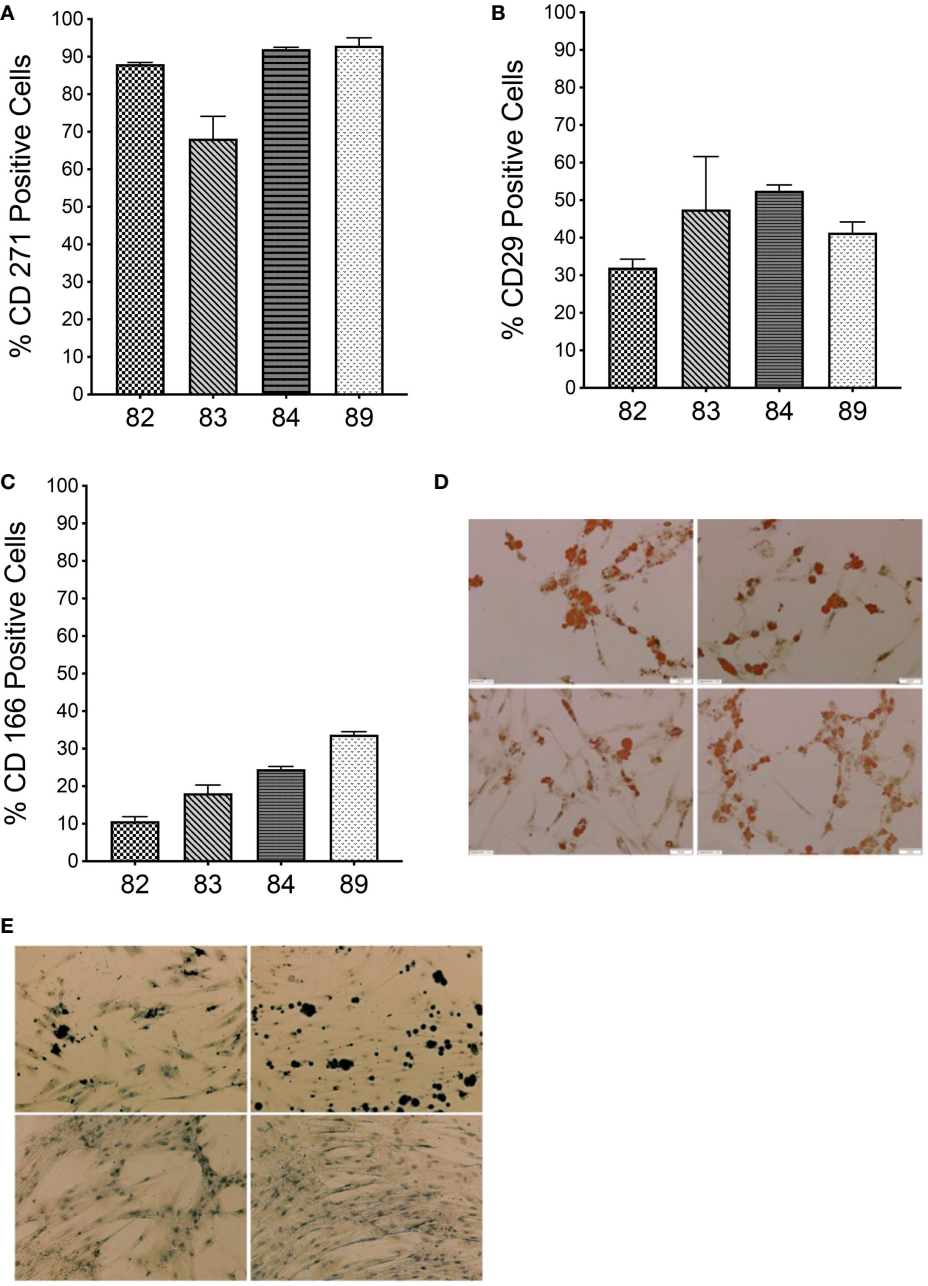
Characterization of autologous BM-derived MSCs. **(A–C)** Flow cytometric analysis of MSCs isolated from sheep bone marrow for CD271, CD29, and CD166 markers. **(D)** Oil Red O staining of MSCs from each sheep after differentiation into adipogenic lineage. **(E)** von Kossa staining of MSCs from each sheep after differentiation into osteocytic lineage. (n=4 biologic replicates; n=3 technical replicates).

First, we confirmed that MSCs isolated from bone marrow of each sheep (n=4) possessed the appropriate phenotype and differentiative potential to be considered MSCs. By contrast to human MSCs, whose identity can be confirmed using a standardized set of phenotypical markers (24), there is not an antibody combination that has been consistently reported to characterize sheep bone marrow-derived MSCs (25). In this study, we considered cells in culture to be MSCs, if they adhered to plastic, were CD45 negative, more than 70% of the cells expressed CD271, and the cells were able to differentiate into osteoblasts and adipocytes. In this study, bone marrow-derived cells isolated and expanded in culture from each of the four animals were more than 70% positive for CD271 (**Figure 1A**) and were devoid of CD45 (data not shown), establishing that there was no hematopoietic cell contamination. In addition, we also determined the percentage of cells that expressed CD29 and CD166 (26–30), and the percentage of cells expressing CD29 and CD166 is shown in **Figures 1B, C**. When cells were placed in adipogenic induction media, cells from all 4 sheep exhibited lipid vacuoles as early as 1 week after commencing induction and contained Oil Red O-positive lipid droplets by day 10 when the staining was performed, confirming the adipogenic differentiation potential of these sheep MSCs (**Figure 1D**). When the cells were induced under osteogenic conditions, calcium deposits were visible by day 14, confirming the osteocytic differentiation potential of these sheep MSCs (**Figure 1E**). Of note is that cells from animals 84 and 89 had less calcium deposits by day 14 than the other two animals (**Figure 1E**).

### Transduction of sheep bone marrow-derived MSCs

**Figure 2:**
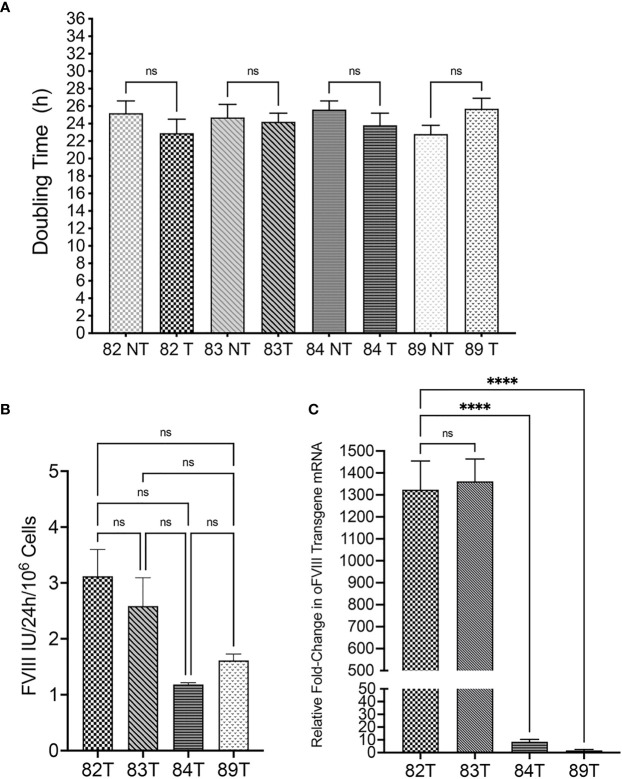
Evaluation of Transduced Sheep Bone Marrow-Derived MSCs. **(A)** Transduction did not exert a statistically significant effect on cell doubling time (n=4 biologic replicates; n=4 technical replicates; p > 0.05). **(B)** FVIII activity in the supernatant of transduced cells was measured by one stage assay (aPTT) to determine the amount of oFVIII protein secreted by each MSCs population; levels of FVIII production differed markedly between the 4 individual sheep, but differences did not reach statistical significance (n=4 biologic replicates; n=3 technical replicates; p > 0.05). **(C)** RT-qPCR quantifying the expression of oFVIII mRNA showed a significant increase in the levels of oFVIII mRNA after transduction, with over a 1000-fold increase in the two MSCs lines that had higher levels of FVIII secretion, but more modest increases in the other two cell lines (n=4 biologic replicates; n=3 technical replicates; ****p < 0.0001). ns, not significant.

After determining that all the isolated cells met the minimum criteria to be considered MSCs, cells were transduced, as detailed in the Materials and Methods, with a replication-defective lentiviral vector encoding a FLAG tag-conjugated oFVIII transgene. Following transduction, population doubling times (PDT) were compared between transduced and non-transduced MSCs. Results demonstrated that transduction did not exert a statistically significant effect on cell growth (**Figure 2A**). FVIII activity in the supernatant of transduced cells was measured to determine the amount of oFVIII protein secreted by each donor’s MSCs (**Figure 2B**). MSCs from each of the 4 sheep produced FVIII protein/activity by a one-stage clotting assay (aPTT) following transduction. Although the levels of FVIII production differed markedly between the 4 individual sheep, differences did not reach statistical significance (p>0.05). Of note is that prior to transduction, none of the sheep MSCs’ supernatant contained detectable amounts of FVIII (data not shown). RT-qPCR was also used to quantify expression of oFVIII mRNA in these cells by determining the relative fold increase in oFVIII transgene RNA in transduced MSCs compared to their respective non-transduced counterparts (**Figure 2C**). These analyses showed a significant increase in the levels of oFVIII mRNA after transduction, with over a 1000-fold increase in the two MSC lines that had higher levels of FVIII secretion, but more modest increases in the other two cell lines. Since all 4 MSC populations were transduced using the same procedure, the same viral stock, and the same multiplicity of infection, we next investigated whether the differences found were due to lower vector copy number (VCN) in those MSCs. MSCs 82, 83, 84, and 89 had VCN of 1.04, 1.98, 0.3, and 0.5 respectively, confirming that the lower production of FVIII was due to variability of transduction efficiency between the different MSCs.

### Autologous oFVIII-FLAG producing MSCs can provide FVIII protein *in vivo* without eliciting an immune response

Autologous oFVIII-FLAG producing MSCs (*MSC-oFVIII-FLAG)* were administered in a single dose, to the respective animals, using an ultrasound-guided IP injection. This route of delivery was selected based on our previous studies showing that IP administration of haploidentical cells resulted in phenotypic correction of sheep with hemophilia A (21). Although the cells produced different amounts of *oFVIII-FLAG*, the cell dose was calculated to provide 60IU/kg/24h of FVIII in all animals. Thus, animals 82, 83, 84, and 89 received 1.27x10^9^, 1.9 x10^9^, 5.4 x10^9^, and 3.7 x10^9^ oFVIII-FLAG producing MSCs, respectively.

**Figure 3:**
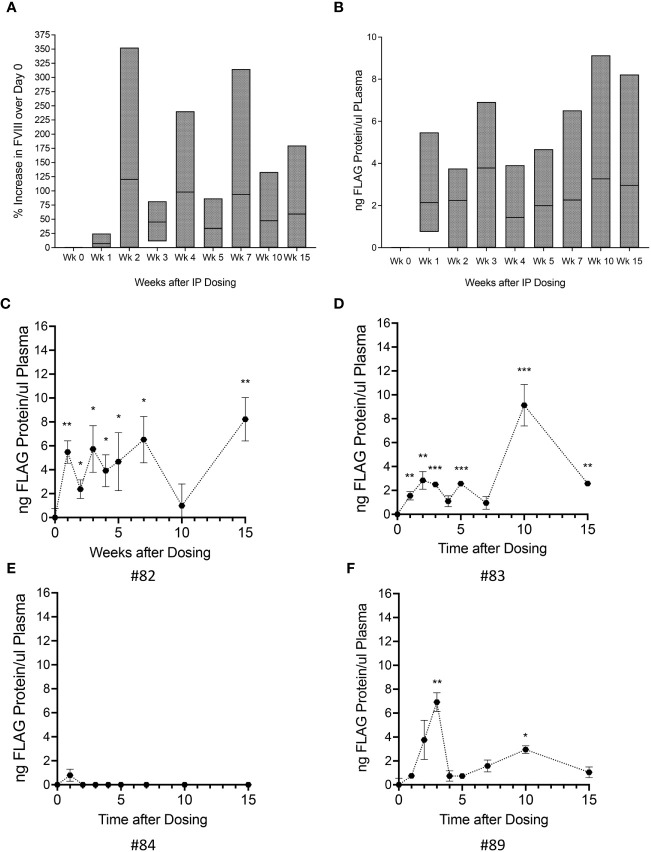
Administration of Autologous oFVIII-FLAG Producing MSCs Provide FVIII Protein in vivo. **(A)** Factor VIII activity measured in the plasma of all animals at weekly intervals after treatment with the autologous MSC-oFVIII-FLAG; the percent increase in FVIII activity over day 0 for each animal was calculated at each time point and the min to max increase in FVIII activity in the group of animals is shown (n=4 biological replicates). **(B)** Min to max levels oFVIII-FLAG protein in the plasma of animals after treatment measured by FLAG-specific ELISA (n=4 biological replicates, n=3 technical replicates). **(C–F)** Levels oFVIII-FLAG protein in the plasma of each individual animal measured by FLAG-specific ELISA (n=3) *p < 0.05, **p < 0.005, ***p < 0.0005 compared to week 0.

We first investigated whether FVIII-FLAG protein was present in plasma and was functional. FVIII activity levels were determined using aPTT at day 0 and weekly thereafter. The percent increase in FVIII activity over day 0 for each animal (n=4) was calculated at each time point, and the overall group increase in FVIII activity is shown in **Figure 3A**.

Having determined that there was an increase in FVIII activity in the plasma of these animals, and because the oFVIII transgene contained a FLAG tag that allowed us to precisely quantitate the circulating levels of oFVIII-FLAG protein derived from the infused cells, we used a FLAG-specific ELISA to quantify the tagged protein. **Figure 3B** depicts the overall group levels of FVIII-FLAG protein in the transplanted animals (n=4) at the different weeks post-transplant.

The presence of FLAG protein in the plasma of each individual animal is also shown in **Figures 3C-F**. Three out of 4 animals had significantly elevated levels of oFVIII-FLAG protein in circulation. In these animals, the levels of oFVIII-FLAG protein varied over time, but this variation did not seem related to time post-administration, as the maximal plasma levels of FLAG protein in one of the animals (animal #82; 8.2 ng/μL) was observed at 15 weeks after transplantation (**Figure 3C**) and in another (animal #83; 9.1 ng/μL) occurred at 10 weeks after transplantation (**Figure 3D**). These data demonstrate that, overall, the cell therapy could be effective in 75% of subjects and provide enough FVIII protein to significantly increase plasma FVIII levels for at least 15 weeks after administration.

**Figure 4:**
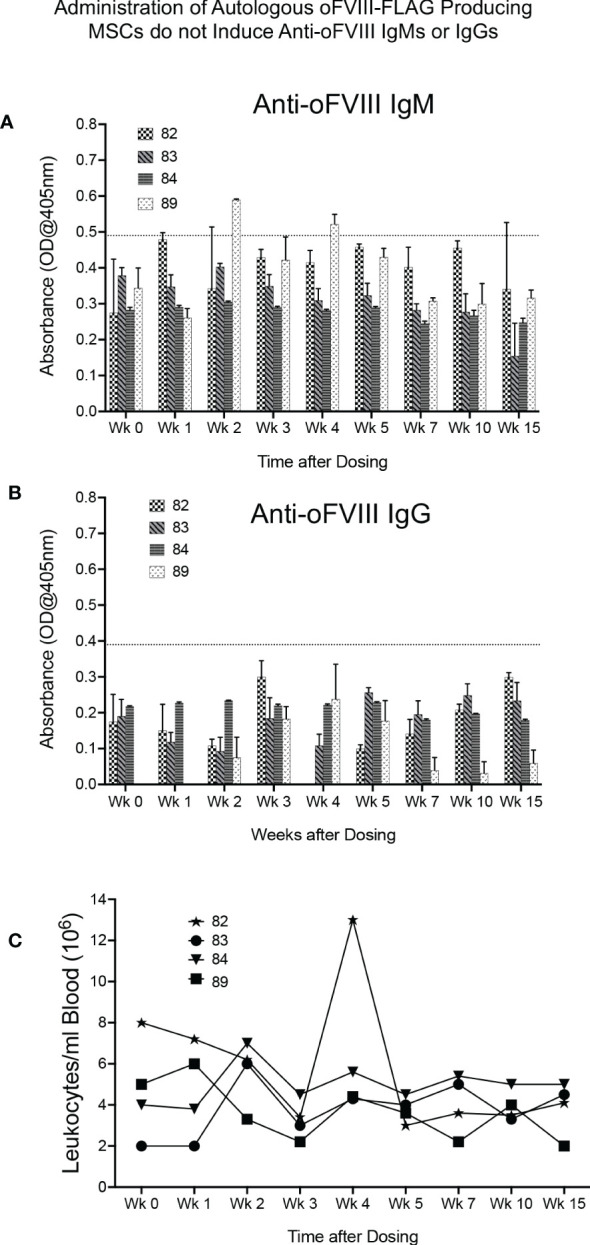
Administration of Autologous oFVIII-FLAG Producing MSCs do not Elicit an Immune Response or cause Noticeable Alterations in White Blood Cell Counts **(A)** Detection of anti-oFVIII IgMs in serum at the lowest dilution of 1:20, for each animal, at each timepoint, measured by ELISA (based on the background observed in a panel of negative control sheep, only signals above 0.49 were deemed to be positive) (n=3). **(B)** Detection of anti-oFVIII IgGs in serum (1:20 dilution) for each animal at each timepoint, measured by ELISA. Based on the background observed in a panel of negative control sheep, only signals above 0.3 were deemed to be positive (n=3). **(C)** White blood cell counts/ml of blood in each animal prior to and for 15 weeks after injections of autologous MSCs.

We next determined whether an immune response to the vector-encoded oFVIII might be responsible, at least in part, for the variation in plasma oFVIII-FLAG levels among the 4 sheep that received the autologous MSC-based treatment, as anti-FVIII antibodies may prevent FVIII function or increase its clearance from circulation. Quantification of anti-FVIII IgM and IgG antibodies was performed using a FVIII-specific ELISA. As can be seen in **Figure 4A**, only one animal developed a transient IgM antibody with a titer of 1:20 at 2- and 4-weeks post-product administration, which did not seem to impact the levels of oFVIII-FLAG in the plasma of that animal at those time points (**Figure 3F**). Of note is that none of the animals developed anti-FVIII IgGs antibodies at any timepoint following transplantation (**Figure 4B**). In addition, we also evaluated whether administration of *MSC-oFVIII-FLAG* would have an effect on white blood cell counts. As can be seen in **Figure 4C**, no significant differences were seen from day 0 to week 15.

### Quantification of autologous MSC-oFVIII-FLAG engraftment in multiple tissues by RT-qPCR

**Figure 5:**
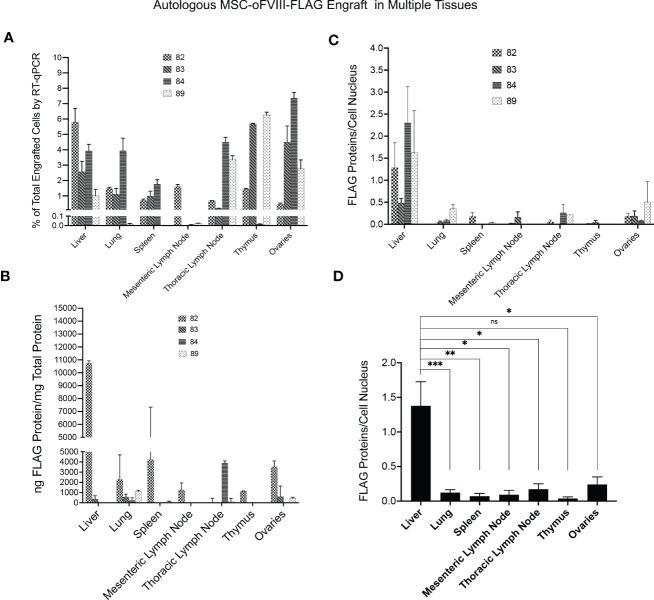
Quantification of Autologous MSC-oFVIII-FLAG Engraftment in Multiple Tissues by RT-qPCR, FLAG-specific ELISA, and Immunohistochemistry. **(A)** RT-qPCR was performed (n=3/organ/animal) using FLAG-tagged oFVIII specific primers on RNA isolated from different organs, and the percentage of MSC-oFVIII-FLAG engraftment was extrapolated using a standard curve prepared with RNA isolated from different percentages of MSC-oFVIII-FLAG mixed with sheep non-transduced MSCs. MSC-oFVIII-FLAG engrafted at detectable levels in each animal in every tissue examined. **(B)** Levels of oFVIII-FLAG protein in each tissue were determined by performing FLAG-specific ELISA on tissue homogenates, and the highest levels of oFVIII-FLAG protein were found in the liver of the treated animals (n=4 biologic replicates; n=3 technical replicates). **(C)** Immunohistochemistry with an antibody to the FLAG-tag protein was also performed in the various tissues to confirm the presence of oFVIII-FLAG in the different tissues; particle analysis in imageJ was used to quantify the total number of cells and the amount of FLAG protein to determine the quantity of FLAG protein per cell, and these analyses showed a pattern of engraftment similar to that found by ELISA. **(D)** Levels of oFVIII-FLAG protein were significantly higher in the liver of the recipients than any other tissue (n=4 biologic replicates; n=3 technical replicates; *p < 0.05, **p < 0.005, ***p < 0.0005). ns, not significant.

Having established that IP transplantation of autologous MSCs producing oFVIII-FLAG, in the absence of any pre-conditioning, resulted in the long-term elevation of plasma FVIII activity, we next performed studies to determine whether these transplanted MSCs engrafted and, if so, to define the sites of engraftment. To accomplish this objective, RNA was isolated from the tissues of the recipients at euthanasia and RT-qPCR was performed with primers to the FLAG-tagged oFVIII. To generate a standard curve to enable quantitation of levels of engrafted *MSC-oFVIII-FLAG* in each tissue, RT-qPCR was also performed on RNA isolated from samples consisting of different percentages of transduced and non-transduced sheep bone marrow MSCs. CT values obtained from each of the tissues were then extrapolated into percentages of engrafted *MSC-oFVIII-FLAG* using the standard curve. As can be seen in **Figure 5A**, autologous *MSC-oFVIII-FLAG* engrafted at detectable levels in each animal in every tissue examined. The highest levels of consistent engraftment were observed in the liver, with every animal having at least 1% engraftment, and animal 82 exhibiting 5.8% engraftment in this tissue. Most animals also exhibited engraftment in the lung, thymus, spleen, and, surprisingly, the ovaries, with animal 84 exhibiting 7.4% engraftment in this tissue – the highest engraftment seen in any tissue from any animal in this study.

The transplanted cells did not engraft at appreciable levels within the mesenteric lymph nodes in any of the animals, with only one animal exhibiting engraftment above 0.1% in this tissue. Among the other tissues analyzed, we were unable to discern a pattern or correlation between which tissues showed engraftment in each animal, as some animals exhibited high levels of engraftment within a given tissue, while other animals exhibited very low levels of engraftment within that same tissue (**Figure 5A**).

### Autologous oFVIII-FLAG producing MSCs engraft in multiple tissues and produce FVIII protein

Next, the levels of oFVIII-FLAG protein in each tissue were determined by performing FLAG-specific ELISA on tissue homogenates (**Figure 5B**). Animal 82 had the highest levels of oFVIII-FLAG protein in every tissue but the thymus, which correlates well with the high levels of FVIII activity and oFVIII-FLAG protein that were seen in the plasma of this animal. The liver of animal 82 also had the highest levels of oFVIII-FLAG protein with 10,725ng oFVIII-FLAG protein/mg total protein, demonstrating that the increased engraftment in that tissue resulted in high levels of oFVIII-FLAG protein production. The other 3 animals all had lower, but still detectable, levels of oFVIII-FLAG protein in some tissues, but oFVIII-FLAG levels were below the limit of detection in other tissues. Apart from animal 82, the highest level of oFVIII-FLAG protein was detected in the thoracic lymph node of animal 84, which contained 3,912 ng oFVIII-FLAG protein/mg total protein. This value is 29 times higher than that seen in the thoracic lymph node of the animal exhibiting the second highest amount of protein in this tissue. This high level of oFVIII-FLAG protein in the thoracic lymph node of animal 84 correlates with the high levels of engraftment observed in this tissue. The lung was the only tissue in which oFVIII-FLAG protein could be detected in all four animals with this tissue-based ELISA. In addition, three animals (82, 83, and 89) displayed oFVIII-FLAG protein in their ovaries and thoracic lymph node, two animals (82 and 83) demonstrated oFVIII-FLAG protein in their liver and spleen, but only animal 82 harbored oFVIII-FLAG protein in its mesenteric lymph node and thymus (**Figure 5B**).

**Figure 6:**
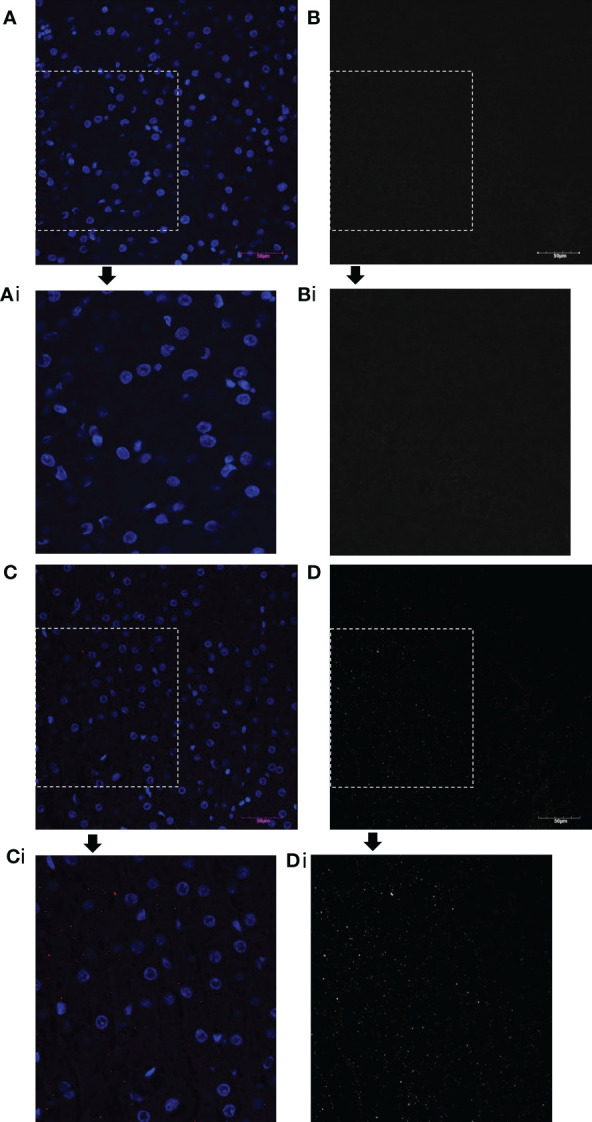
oFVIII-FLAG Immunohistochemistry Images of Liver Sections from a Control and a Treated Animal. **(A)** Representative images of the IHC staining of the liver from a control non-transplanted animal with an antibody to the FLAG-tag protein; **(Ai)** amplified inset area from A. **(B, Bi)** Same image as A and Ai but in black and white for better visualization after extracting the red channel and converting to grayscale in Photoshop. **(C)** Representative image of the IHC staining of the liver of treated animal with an antibody to the FLAG-tag protein (red); **(Ci)** amplified inset area from C. **(D, Di)** Same image as C and Ci but in black and white for better visualization after extracting the red channel and converting to grayscale in Photoshop. All images were acquired with an Olympus BX63 microscope Olympus with a 20x objective.

Immunohistochemistry with an antibody to the FLAG-tag protein was also performed in the various tissues to confirm the presence of oFVIII-FLAG in the different tissues (**Figure 5C**), and these analyses showed a pattern of engraftment similar to that found by ELISA. In addition, data showed that, overall, the levels of oFVIII-FLAG protein were significantly higher in the liver of the recipients than any other tissue (p<0.05) (**Figure 5D**). Representative images of the IHC staining of the liver from a control animal (**Figures 6 A, B**) and animal 82 (**Figures 6 C, D**) demonstrate the presence of FLAG protein (in red) throughout the liver of the treated animal. In addition, insets indicated by the letter “i” demonstrate detail of the highlighted area. Black and white images of FLAG-tag protein staining (red channel) in the control and animal 82 are also included for better visualization.

## Discussion

As a monogenic disease, HA is an ideal candidate for correction by cell and/or gene therapy, as addition of a functional FVIII gene could provide the missing FVIII protein. The potential for using cells as a platform to deliver FVIII to treat HA has been demonstrated in several studies using murine models (31–36) which have resulted in the development of 2 clinical trials. In these, autologous HSC transduced with lentiviral vectors expressing engineered forms of FVIII are infused into the patient after reduced intensity conditioning treatment. Nevertheless, patients undergoing HSC transplantation are at risk of severe complications (37), and conditioning regimens not only contribute to late mortality but also cause substantial morbidity and impair long-term health-related quality of life in transplanted patients (38). Thus, the ability to use cells that can engraft and efficiently deliver FVIII in the absence of conditioning would be a major paradigm shift and would expand the pool of eligible patients.

Studies transplanting endothelial colony-forming cells and placenta-derived stromal cells (39), or liver sinusoidal endothelial cells (LSEC), derived from normal human induced pluripotent stem cells (iPSC) (31), or even HA patient-derived iPSC engineered *via* CRISPR/Cas9 to express functional FVIII (32), resulted in clinical correction of HA mice. While these studies highlight the potential of cell-based therapies for treating HA, the study using autologous cells provides critical proof-of-concept that cells from patients with HA can be modified to produce functional FVIII protein and transplanted to correct HA. Despite the immense promise of iPSC-based therapies, however, the use of iPSC will require any therapy using their cellular derivatives to undergo exhaustive, long-term preclinical safety testing to ensure that the clinical product is completely safe (40). In addition, an important issue that should be addressed regarding safety of any cell therapy, especially MSCs, is the potential induction of pro-thrombotic events due to the expression of tissue factor (TF) (41, 42).

In the present report, we performed studies in a translational large animal (sheep) model, whose size is similar to that of humans, thus allowing the evaluation of the efficacy and safety of therapies without the need for later scale-up, and we demonstrated that autologous BM-derived MSCs can be isolated, efficiently engineered *in vitro* with a lentiviral vector to produce and secrete functional FVIII, and subsequently be expanded to clinically relevant numbers. Moreover, we show that, upon intraperitoneal injection into adult animals, in the absence of any form of pre-conditioning, these cells distribute throughout the body, as evidenced by the presence of oFVIII-FLAG in several different organs of the recipient animals. MSCs persisted in multiple tissues, where they stably produce and secrete FVIII into the circulation. Although we were unable to test if the sheep MSCs expressed TF due to lack of a reliable sheep-specific antibody, none of the animals that received the therapy had any signs of thrombotic events. Nevertheless, expression of TF in the context of hemophilia would not be deemed problematic, as the TF : FVIIa complex of the extrinsic pathway initiates blood coagulation by activating both FX and FIX, and there are currently products on the market that use recombinant activated factor VII (rFVIIa) to treat and/or prevent hemorrhagic complications in persons with hemophilia with inhibitors (43). Data also demonstrated that none of the animals that received autologous MSC-based treatment developed IgG antibodies to FVIII, suggesting this approach may avoid inhibitor formation, the most serious complication plaguing current protein-based HA treatment. An important caveat, however, is that the present studies were performed in healthy/wild-type animals with normal levels of endogenous oFVIII. In a previous pilot study performed in 2 HA animals, we showed that the IP administration of haploidentical BM-derived MSCs expressing high levels of FVIII phenotypically corrected the animals but led to high titer inhibitors (21). Several key differences exist between these two studies, however, that likely account for this apparent discrepancy. First, the HA animals had received multiple infusions of a variety of human FVIII products in an effort to control active bleeds and crippling hemarthroses prior to being transplanted with the FVIII-expressing MSCs. As a result, these animals already had low-titer inhibitors at the time of MSC infusion. Of note, is that in the present studies cells secreting oFVIII-FLAG were only injected once, and not multiple times. Nevertheless, at least 3 of the animals had oFVIII-FLAG protein circulating in their plasma for 15 weeks and as such, if the product was immunogenic, it could have induced anti-oFVIII-FLAG antibodies. A second key difference is that the HA animals were transplanted with paternal (haploidentical), not autologous MSCs. A third critical difference between these studies is that the HA animals were transplanted with MSCs that had been transduced with lentiviral vectors encoding GFP and porcine FVIII, as the cDNA for oFVIII was not available at the time this earlier study was performed. As such, the use of a xenogeneic FVIII transgene and the inclusion of the highly immunogenic GFP reporter (44) also likely played a major role in the robust anti-FVIII response we observed in these HA animals (21). In the present study, we sought to eliminate these potential immune triggers by using autologous MSCs that had been modified to express B domain-deleted (due to vector size constraints) oFVIII. To enable tracking of the transplanted autologous cells and detection of the vector-derived oFVIII protein in the circulation of normal (non-HA) animals, we avoided the use of a fluorescent reporter and instead opted to engineer the oFVIII transgene to contain a FLAG tag, one of the few protein tags that has been shown to be non-immunogenic, even with repeated challenge (45). While definitive evidence of clinical efficacy will require future studies in HA animals, the results described herein provide critical proof-of-concept for the suitability of autologous BM-derived MSCs as cellular vehicles to deliver FVIII and provide long-term (at least 15 weeks) treatment for HA.

It is of note that all animals received appropriate numbers of autologous MSCs to provide a specific dose of 60IU/kg/24h, still not all animals exhibited increased levels of oFVIII activity and/or sustained levels of oFVIII-FLAG protein in their plasma. The reasons for this animal-to-animal variability are presently not understood, as it is not clear why some of the BM-derived MSCs were more resistant to transduction than others. This underscores the need for caution when using personalized therapies and the need to establish safety and efficacy parameters that withstand biological variability. In addition, the use of autologous cells in persons with HA may be complicated by the need to harvest bone marrow. Although the procedure is deemed extremely safe, it is still invasive, and for those at risk of bleeding, it may pose a higher risk of complications. Also, because the autologous MSCs are transduced in order to overexpress FVIII, each personalized product would have to undergo testing all the parameters pertaining to safety and efficacy, as per the release criteria established for the product(s), including potential insertional mutagenesis. To this end, an off-the-shelf product may be as safe, and ultimately be more affordable, than a personalized therapy, as only a single product needs to be manufactured and tested.

This study also shows that the high levels of oFVIII-FLAG production/activity *in vivo* correlated with high VCN, oFVIII mRNA levels, and the *in vitro* FVIII activity on a per cell basis prior to transplant. *In vitro* and *in vivo* data correlated quite well in the animal that exhibited low/undetectable oFVIII-FLAG protein in its plasma and exhibited no increase in plasma oFVIII-FLAG following transplantation with its autologous MSCs, as this animal displayed the lowest VCN, *in vitro* FVIII activity, and oFVIII mRNA levels of the four animals. Thus, it is possible that increasing the cell dose to provide the desired FVIII output is not enough to obtain the same therapeutic effect. Also interesting is that this animal had very low levels of engraftment in most of its tissues, with the exception of the thoracic lymph node, where high levels of oFVIII-FLAG protein were also observed. Since this animal did not develop anti-oFVIII-FLAG antibodies, it is not clear if presentation of oFVIII-FLAG to immune cells at this site could have contributed to low levels of engraftment.

In other recipients, autologous oFVIII-producing MSCs engrafted in multiple tissues following IP transplantation. Of potential concern would be the presence of oFVIII-FLAG in the ovaries of these animals. It is possible that the IP route of administration led to engraftment of the cells in this organ; nevertheless, MSCs are adult cells, and although studies have reported tissue-specific-like differentiation of MSCs *in vivo* when transplanted *in utero* (46, 47), it would be rather difficult for these cells to fully differentiate into *bona fide* germline cells.

The tissue that consistently exhibited the highest levels of engraftment and of oFVIII-FLAG expression in all 4 animals was the liver. Since the liver is the primary site of native FVIII expression in the body, engraftment of the transplanted MSCs and their expression of vector-driven oFVIII in this tissue should provide the most physiologically accurate expression and secretion of FVIII, as local signaling may promote FVIII production and release of the protein into the circulation. Of significance to the high-level engraftment and expression of FVIII within the liver is also the unique immunological milieu of the liver. There have been numerous reports that expression of exogenous genes and their resultant proteins within the liver has the ability to promote induction of tolerance to the expressed protein, likely due, at least in part, to the induction of Tregs (48–68). Engraftment within this tissue would thus be ideal for treating HA, as the liver is the predominant site of FVIII synthesis in the body (69) and expression of the perceived foreign protein FVIII within the liver may promote tolerance and thereby avoid inhibitor induction. Future studies to identify means of further promoting liver engraftment could improve upon this cellular therapy, as this would enable a lower dose of cells to be administered while maintaining sufficient levels of FVIII to correct bleeding diathesis.

In conclusion, the present studies provide compelling evidence that lentiviral vector-transduced autologous BM-derived MSCs are well-suited as cellular vehicles for safely delivering FVIII and achieving therapeutic plasma FVIII levels without eliciting an immune response. Nevertheless, translation of this approach to the clinic will require studies in HA animals to validate the true therapeutic potential of this autologous therapy. Also, it would be necessary to extend the testing of the animals to several years to determine how long the transduced cells are able to provide therapeutic levels of FVIII. These studies also did not investigate whether IP is the only possible route of administration. If the therapy proves to be long-lasting or curative, then IP administration would be acceptable, but if the therapy is recurring, then other less invasive routes of administration such as IV should be utilized. These studies were conducted in adult sheep; thus, one should also determine the ideal age at which to recommend this procedure, as age could impact levels of cell engraftment. Since *MSC-oFVIII-FLAG* were injected per kg of body weight to provide 60IU/kg/24hr, it would be pertinent to find out if the therapy is administered at an early age whether therapeutic levels of FVIII can be maintained despite the increase in body size. Finally, it would be interesting to to know whether the proposed therapy would still work if FVIII inhibitory antibodies are already present in the recipient.

We hope that the present studies can serve as a springboard to studies that ultimately will pave the way for clinical development of this straightforward and highly promising HA treatment.

## Data availability statement

The raw data supporting the conclusions of this article will be made available by the authors, without undue reservation.

## Ethics statement

The animal study was reviewed and approved by Wake Forest University Institutional Animal Care and Use Committee.

## Author contributions

BT, MR, HM, executed experiments, data analysis and interpretation. SL, RC, JO provided technical expertise. AA provided reagents and experimental feedback. BT drafted manuscript. GA-P and CP conception and experimental design, supervised experiments, performed data analysis and interpretation, wrote final version of the manuscript, and secured funding. GA-P and CD contributed equally to his work.

## Funding

This work was supported by NIH, NHLBI, HL130856, HL135853. BT is supported by a T32 pre-doctoral fellow position NIH NIBIB 2T32EB014836-06A. MR and GA-P are the recipients of a fellow/mentor HHMI Gilliam Graduate Fellowship grant.

## Acknowledgments

We would like to thank Wake Forest Baptist Health Special Hematology Laboratory for their excellent technical support and the performance of FVIII testing, and the Wake Forest ARP for the clinical care of the animals.

## Conflict of interest

The authors declare that the research was conducted in the absence of any commercial or financial relationships that could be construed as a potential conflict of interest.

## Publisher’s note

All claims expressed in this article are solely those of the authors and do not necessarily represent those of their affiliated organizations, or those of the publisher, the editors and the reviewers. Any product that may be evaluated in this article, or claim that may be made by its manufacturer, is not guaranteed or endorsed by the publisher.

## References

[1] Giordano A Galderisi U Marino IR. From the laboratory bench to the patient’s bedside: An update on clinical trials with mesenchymal stem cells. J Cell Physiol (2007) 211(1):27–35. doi: 10.1002/jcp.20959 17226788

[2] Ringden O Moll G Gustafsson B Sadeghi B. Mesenchymal stromal cells for enhancing hematopoietic engraftment and treatment of graft-Versus-Host disease, hemorrhages and acute respiratory distress syndrome. Front Immunol (2022) 13:839844. doi: 10.3389/fimmu.2022.839844 35371003 PMC8973075

[3] Almeida-Porada G Atala AJ Porada CD. Therapeutic mesenchymal stromal cells for immunotherapy and for gene and drug delivery. Mol Ther Methods Clin Dev (2020) 16:204–24. doi: 10.1016/j.omtm.2020.01.005 PMC7012781 32071924

[4] Di Nicola M Carlo-Stella C Magni M Milanesi M Longoni PD Matteucci P. Human bone marrow stromal cells suppress T-lymphocyte proliferation induced by cellular or nonspecific mitogenic stimuli. Blood (2002) 99(10):3838–43. doi: 10.1182/blood.V99.10.3838 11986244

[5] Joswig A-J Mitchell A Cummings KJ Levine GJ Gregory CA Smith R. Repeated intra-articular injection of allogeneic mesenchymal stem cells causes an adverse response compared to autologous cells in the equine model. Stem Cell Res Ther (2017) 8(1):42. doi: 10.1186/s13287-017-0503-8 28241885 PMC5329965

[6] Isakova IA Lanclos C Bruhn J Kuroda MJ Baker KC Krishnappa V. Allo-reactivity of mesenchymal stem cells in rhesus macaques is dose and haplotype dependent and limits durable cell engraftment in vivo. PloS One (2014) 9(1):e87238. doi: 10.1371/journal.pone.0087238 24489878 PMC3906169

[7] Franchini M Mannucci PM. Hemophilia a in the third millennium. Blood Rev (2013) 27(4):179–84. doi: 10.1016/j.blre.2013.06.002 23815950

[8] El-Akabawy N Rodriguez M Ramamurthy R Rabah A Trevisan B Morsi A. Defining the optimal FVIII transgene for placental cell-based gene therapy to treat hemophilia a. Mol Ther - Methods Clin Dev (2020) 17:465–77. doi: 10.1016/j.omtm.2020.03.001 PMC7109377 32258210

[9] Kaufman RJ Pipe SW Tagliavacca L Swaroop M Moussalli M. Biosynthesis, assembly and secretion of coagulation factor VIII. Blood Coagul Fibrinolysis. (1997) 8 Suppl 2:S3–14. doi: 10.1111/jth.14204 9607108

[10] Saenko EL Ananyeva NM Shima M Hauser CA Pipe SW. The future of recombinant coagulation factors. J Thromb haemostasis: JTH. (2003) 1(5):922–30. doi: 10.1046/j.1538-7836.2003.00196.x 12871357

[11] Vander Kooi A Wang S Fan MN Chen A Zhang J Chen CY. Influence of n-glycosylation in the a and c domains on the immunogenicity of factor VIII. Blood Adv (2022) 6(14):4271–82. doi: 10.1182/bloodadvances.2021005758 PMC9327553 35511725

[12] Qu J Ma C Xu XQ Xiao M Zhang J Li D. Comparative glycosylation mapping of plasma-derived and recombinant human factor VIII. PloS One (2020) 15(5):e0233576. doi: 10.1371/journal.pone.0233576 32442215 PMC7244179

[13] Ito J Baldwin WH Cox C Healey JF Parker ET Legan ER. Removal of single-site n-linked glycans on factor VIII alters binding of domain-specific monoclonal antibodies. J Thromb haemostasis: JTH. (2022) 20(3):574–88. doi: 10.1111/jth.15616 PMC8885965 34863021

[14] Delignat S Rayes J Dasgupta S Gangadharan B Denis CV Christophe OD. Removal of mannose-ending glycan at Asn(2118) abrogates FVIII presentation by human monocyte-derived dendritic cells. Front Immunol (2020) 11:393. doi: 10.3389/fimmu.2020.00393 32273875 PMC7117063

[15] Cormier M Batty P Tarrant J Lillicrap D. Advances in knowledge of inhibitor formation in severe haemophilia a. Br J Haematol (2020) 189(1):39–53. doi: 10.1111/bjh.16377 32064603

[16] Sandberg H Kannicht C Stenlund P Dadaian M Oswaldsson U Cordula C. Functional characteristics of the novel, human-derived recombinant FVIII protein product, human-cl rhFVIII. Thromb Res (2012) 130(5):808–17. doi: 10.1016/j.thromres.2012.08.311 23010293

[17] Valentino LA Negrier C Kohla G Tiede A Liesner R Hart D. The first recombinant FVIII produced in human cells–an update on its clinical development programme. Haemophilia (2014) 20 Suppl 1:1–9. doi: 10.1111/hae.12322 24330348

[18] Lissitchkov T Klukowska A Pasi J Kessler CM Klamroth R Liesner RJ. Efficacy and safety of simoctocog alfa (Nuwiq(R)) in patients with severe hemophilia a: a review of clinical trial data from the GENA program. Ther Adv Hematol (2019) 10:2040620719858471. doi: 10.1177/2040620719858471 31263528 PMC6595650

[19] Liesner RJ Abraham A Altisent C Belletrutti MJ Carcao M Carvalho M. Simoctocog Alfa (Nuwiq) in previously untreated patients with severe haemophilia a: Final results of the NuProtect study. Thromb Haemost. (2021) 121(11):1400–8. doi: 10.1055/s-0040-1722623 PMC8570909 33581698

[20] Pocock SJ. Clinical trials: a practical approach. Chichester West Sussex; New York: Wiley (1983). p. 266.

[21] Porada C Sanada C Kuo E Colletti E Moot R Doering C. Phenotypic correction of hemophilia a by postnatal intraperitoneal transplantation of FVIII-expressing MSC. Blood (2010) 116(21):249–. doi: 10.1182/blood.V116.21.249.249 PMC3220800 21906573

[22] Soland MA Bego MG Colletti E Porada CD Zanjani ED St Jeor S. Modulation of human mesenchymal stem cell immunogenicity through forced expression of human cytomegalovirus us proteins. PloS One (2012) 7(5):e36163. doi: 10.1371/journal.pone.0036163 22666319 PMC3364258

[23] Lytle AM Brown HC Paik NY Knight KA Wright JF Spencer HT. Effects of FVIII immunity on hepatocyte and hematopoietic stem cell-directed gene therapy of murine hemophilia a. Mol Ther Methods Clin Dev (2016) 3:15056. doi: 10.1038/mtm.2015.56 26909355 PMC4750467

[24] Dominici M Le Blanc K Mueller I Slaper-Cortenbach I Marini F Krause D. Minimal criteria for defining multipotent mesenchymal stromal cells. Int Soc Cell Ther position statement. Cytother (2006) 8(4):315–7. doi: 10.1080/14653240600855905 16923606

[25] Music E Futrega K Doran MR. Sheep as a model for evaluating mesenchymal stem/stromal cell (MSC)-based chondral defect repair. Osteoarthritis Cartilage. (2018) 26(6):730–40. doi: 10.1016/j.joca.2018.03.006 29580978

[26] Lv FJ Tuan RS Cheung KM Leung VY. Concise review: the surface markers and identity of human mesenchymal stem cells. Stem Cells (2014) 32(6):1408–19. doi: 10.1002/stem.1681 24578244

[27] Li H Ghazanfari R Zacharaki D Lim HC Scheding S. Isolation and characterization of primary bone marrow mesenchymal stromal cells. Ann N Y Acad Sci (2016) 1370(1):109–18. doi: 10.1111/nyas.13102 27270495

[28] Brinkhof B Zhang B Cui Z Ye H Wang H. ALCAM (CD166) as a gene expression marker for human mesenchymal stromal cell characterisation. Gene X. (2020) 5:100031. doi: 10.1016/j.gene.2020.100031 32550557 PMC7285916

[29] Khan MR Chandrashekran A Smith RK Dudhia J. Immunophenotypic characterization of ovine mesenchymal stem cells. Cytometry A. (2016) 89(5):443–50. doi: 10.1002/cyto.a.22849 27077783

[30] Jessop HL Noble BS Cryer A. The differentiation of a potential mesenchymal stem cell population within ovine bone marrow. Biochem Soc Trans (1994) 22(3):248S. doi: 10.1042/bst022248s 7821511

[31] Gage BK Merlin S Olgasi C Follenzi A Keller GM. Therapeutic correction of hemophilia a by transplantation of hPSC-derived liver sinusoidal endothelial cell progenitors. Cell Rep (2022) 39(1):110621. doi: 10.1016/j.celrep.2022.110621 35385743

[32] Son JS Park CY Lee G Park JY Kim HJ Kim G. Therapeutic correction of hemophilia a using 2D endothelial cells and multicellular 3D organoids derived from CRISPR/Cas9-engineered patient iPSCs. Biomaterials (2022) 283:121429. doi: 10.1016/j.biomaterials.2022.121429 35217482

[33] Doering CB Gangadharan B Dukart HZ Spencer HT. Hematopoietic stem cells encoding porcine factor VIII induce pro-coagulant activity in hemophilia a mice with pre-existing factor VIII immunity. Mol Ther (2007) 15(6):1093–9. doi: 10.1038/sj.mt.6300146 17387335

[34] Ide LM Iwakoshi NN Gangadharan B Jobe S Moot R McCarty D. Functional aspects of factor VIII expression after transplantation of genetically-modified hematopoietic stem cells for hemophilia a. J Gene Med (2010) 12(4):333–44. doi: 10.1002/jgm.1442 20209485

[35] Shi Q Fahs SA Wilcox DA Kuether EL Morateck PA Mareno N. Syngeneic transplantation of hematopoietic stem cells that are genetically modified to express factor VIII in platelets restores hemostasis to hemophilia a mice with preexisting FVIII immunity. Blood (2008) 112(7):2713–21. doi: 10.1182/blood-2008-02-138214 PMC2556608 18495954

[36] Tiede A Eder M von Depka M Battmer K Luther S Kiem HP. Recombinant factor VIII expression in hematopoietic cells following lentiviral transduction. Gene Ther (2003) 10(22):1917–25. doi: 10.1038/sj.gt.3302093 14502221

[37] Khaddour K Hana CK Mewawalla P. Hematopoietic stem cell transplantation. Treasure Island (FL: StatPearls (2022). 30725636

[38] Clavert A Peric Z Brissot E Malard F Guillaume T Delaunay J. Late complications and quality of life after reduced-intensity conditioning allogeneic stem cell transplantation. Biol Blood Marrow Transplant (2017) 23(1):140–6. doi: 10.1016/j.bbmt.2016.10.011 27751934

[39] Gao K Kumar P Cortez-Toledo E Hao D Reynaga L Rose M. Potential long-term treatment of hemophilia a by neonatal co-transplantation of cord blood-derived endothelial colony-forming cells and placental mesenchymal stromal cells. Stem Cell Res Ther (2019) 10(1):34. doi: 10.1186/s13287-019-1138-8 30670078 PMC6341603

[40] Jha BS Farnoodian M Bharti K. Regulatory considerations for developing a phase I investigational new drug application for autologous induced pluripotent stem cells-based therapy product. Stem Cells Transl Med (2021) 10(2):198–208. doi: 10.1002/sctm.20-0242 32946199 PMC7848308

[41] Moll G Ignatowicz L Catar R Luecht C Sadeghi B Hamad O. Different procoagulant activity of therapeutic mesenchymal stromal cells derived from bone marrow and placental decidua. Stem Cells Dev (2015) 24(19):2269–79. doi: 10.1089/scd.2015.0120 26192403

[42] Moll G Drzeniek N Kamhieh-Milz J Geissler S Volk HD Reinke P. MSC therapies for COVID-19: Importance of patient coagulopathy, thromboprophylaxis, cell product quality and mode of delivery for treatment safety and efficacy. Front Immunol (2020) 11:1091. doi: 10.3389/fimmu.2020.01091 32574263 PMC7249852

[43] Ciolek AM Arnall J Moore DC Palkimas S Der-Nigoghossian J Dane K. Eptacog beta for bleeding treatment and prevention in congenital hemophilia a and b with inhibitors: A review of clinical data and implications for clinical practice. Ann Pharmacother. (2022) 56(7):831–8. doi: 10.1177/10600280211049394 34595941

[44] Stripecke R Carmen Villacres M Skelton D Satake N Halene S Kohn D. Immune response to green fluorescent protein: implications for gene therapy. Gene Ther (1999) 6(7):1305–12. doi: 10.1038/sj.gt.3300951 10455440

[45] Chiarella P Edelmann B Fazio VM Sawyer AM Marco. A. Antigenic features of protein carriers commonly used in immunisation trials. Biotechnol Letters. (2010) 32:1215–21. doi: 10.1007/s10529-010-0283-z 20431911

[46] Chamberlain J Yamagami T Colletti E Theise ND Desai J Frias A. Efficient generation of human hepatocytes by the intrahepatic delivery of clonal human mesenchymal stem cells in fetal sheep. Hepatology (2007) 46(6):1935–45. doi: 10.1002/hep.21899 17705296

[47] Colletti EJ Airey JA Liu W Simmons PJ Zanjani ED Porada CD. Generation of tissue-specific cells from MSC does not require fusion or donor-to-host mitochondrial/membrane transfer. Stem Cell Res (2009) 2(2):125–38. doi: 10.1016/j.scr.2008.08.002 PMC3969729 19383418

[48] Arnold B. Parenchymal cells in immune and tolerance induction. Immunol Lett (2003) 89(2-3):225–8. doi: 10.1016/S0165-2478(03)00150-0 14556982

[49] Ferber I Schonrich G Schenkel J Mellor AL Hammerling GJ Arnold B. Levels of peripheral T cell tolerance induced by different doses of tolerogen. Science (1994) 263(5147):674–6. doi: 10.1126/science.8303275 8303275

[50] Schonrich G Momburg F Malissen M Schmitt-Verhulst AM Malissen B Hammerling GJ. Distinct mechanisms of extrathymic T cell tolerance due to differential expression of self antigen. Int Immunol (1992) 4(5):581–90. doi: 10.1093/intimm/4.5.581 1627495

[51] Bowen DG McCaughan GW Bertolino P. Intrahepatic immunity: a tale of two sites? Trends Immunol (2005) 26(10):512–7. doi: 10.1016/j.it.2005.08.005 16109501

[52] Herzog RW Dobrzynski E. Immune implications of gene therapy for hemophilia. Semin Thromb Hemost. (2004) 30(2):215–26. doi: 10.1055/s-2004-825635 15118933

[53] Knolle PA Gerken G. Local control of the immune response in the liver. Immunol Rev (2000) 174:21–34. doi: 10.1034/j.1600-0528.2002.017408.x 10807504

[54] Limmer A Knolle PA. Liver sinusoidal endothelial cells: a new type of organ-resident antigen-presenting cell. Arch Immunol Ther Exp (Warsz). (2001) 49 Suppl 1:S7–11. 11603871

[55] Pastore L Morral N Zhou H Garcia R Parks RJ Kochanek S. Use of a liver-specific promoter reduces immune response to the transgene in adenoviral vectors. Hum Gene Ther (1999) 10(11):1773–81. doi: 10.1089/10430349950017455 10446917

[56] Tokita D Ohdan H Onoe T Hara H Tanaka Y Asahara T. Liver sinusoidal endothelial cells contribute to alloreactive T-cell tolerance induced by portal venous injection of donor splenocytes. Transpl Int (2005) 18(2):237–45. doi: 10.1111/j.1432-2277.2004.00045.x 15691278

[57] De Geest BR Van Linthout SA Collen D. Humoral immune response in mice against a circulating antigen induced by adenoviral transfer is strictly dependent on expression in antigen-presenting cells. Blood (2003) 101(7):2551–6. doi: 10.1182/blood-2002-07-2146 12446451

[58] Dobrzynski E Fitzgerald JC Cao O Mingozzi F Wang L Herzog RW. Prevention of cytotoxic T lymphocyte responses to factor IX-expressing hepatocytes by gene transfer-induced regulatory T cells. Proc Natl Acad Sci U S A. (2006) 103(12):4592–7. doi: 10.1073/pnas.0508685103 PMC1450216 16537361

[59] Dobrzynski E Mingozzi F Liu YL Bendo E Cao O Wang L. Induction of antigen-specific CD4+ T-cell anergy and deletion by *in vivo* viral gene transfer. Blood (2004) 104(4):969–77. doi: 10.1182/blood-2004-03-0847 15105293

[60] Follenzi A Battaglia M Lombardo A Annoni A Roncarolo MG Naldini L. Targeting lentiviral vector expression to hepatocytes limits transgene-specific immune response and establishes long-term expression of human antihemophilic factor IX in mice. Blood (2004) 103(10):3700–9. doi: 10.1182/blood-2003-09-3217 14701690

[61] Franco LM Sun B Yang X Bird A Zhang H Schneider A. Evasion of immune responses to introduced human acid alpha-glucosidase by liver-restricted expression in glycogen storage disease type II. Mol Ther (2005) 12(5):876–84. doi: 10.1016/j.ymthe.2005.04.024 16005263

[62] Mingozzi F Liu YL Dobrzynski E Kaufhold A Liu JH Wang Y. Induction of immune tolerance to coagulation factor IX antigen by *in vivo* hepatic gene transfer. J Clin Invest (2003) 111(9):1347–56. doi: 10.1172/JCI200316887 PMC154443 12727926

[63] Mount JD Herzog RW Tillson DM Goodman SA Robinson N McCleland ML. Sustained phenotypic correction of hemophilia b dogs with a factor IX null mutation by liver-directed gene therapy. Blood (2002) 99(8):2670–6. doi: 10.1182/blood.V99.8.2670 11929752

[64] Nathwani AC Davidoff A Hanawa H Zhou JF Vanin EF Nienhuis AW. Factors influencing *in vivo* transduction by recombinant adeno-associated viral vectors expressing the human factor IX cDNA. Blood (2001) 97(5):1258–65. doi: 10.1182/blood.V97.5.1258 11222368

[65] Nathwani AC Davidoff AM Hanawa H Hu Y Hoffer FA Nikanorov A. Sustained high-level expression of human factor IX (hFIX) after liver-targeted delivery of recombinant adeno-associated virus encoding the hFIX gene in rhesus macaques. Blood (2002) 100(5):1662–9. doi: 10.1182/blood-2002-02-0589 12176886

[66] Schiedner G Hertel S Johnston M Dries V van Rooijen N Kochanek S. Selective depletion or blockade of kupffer cells leads to enhanced and prolonged hepatic transgene expression using high-capacity adenoviral vectors. Mol Ther (2003) 7(1):35–43. doi: 10.1016/S1525-0016(02)00017-5 12573616

[67] Zhang J Xu L Haskins ME Parker Ponder K. Neonatal gene transfer with a retroviral vector results in tolerance to human factor IX in mice and dogs. Blood (2004) 103(1):143–51. doi: 10.1182/blood-2003-06-2181 12969967

[68] Ziegler RJ Lonning SM Armentano D Li C Souza DW Cherry M. AAV2 vector harboring a liver-restricted promoter facilitates sustained expression of therapeutic levels of alpha-galactosidase a and the induction of immune tolerance in fabry mice. Mol Ther (2004) 9(2):231–40. doi: 10.1016/j.ymthe.2003.11.015 14759807

[69] Fahs SA Hille MT Shi Q Weiler H Montgomery RR. A conditional knockout mouse model reveals endothelial cells as the principal and possibly exclusive source of plasma factor VIII. Blood (2014) 123(24):3706–13. doi: 10.1182/blood-2014-02-555151 PMC4055921 24705491

