# Supplementary figures and images for: Autologous bone marrow-derived MSCs engineered to express oFVIII-FLAG engraft in adult sheep and produce an effective increase in plasma FVIII levels
Source: Front Immunol. 2022 Dec 2;13:1070476. doi: 10.3389/fimmu.2022.1070476 (PMC9755880; doi:10.3389/fimmu.2022.1070476)

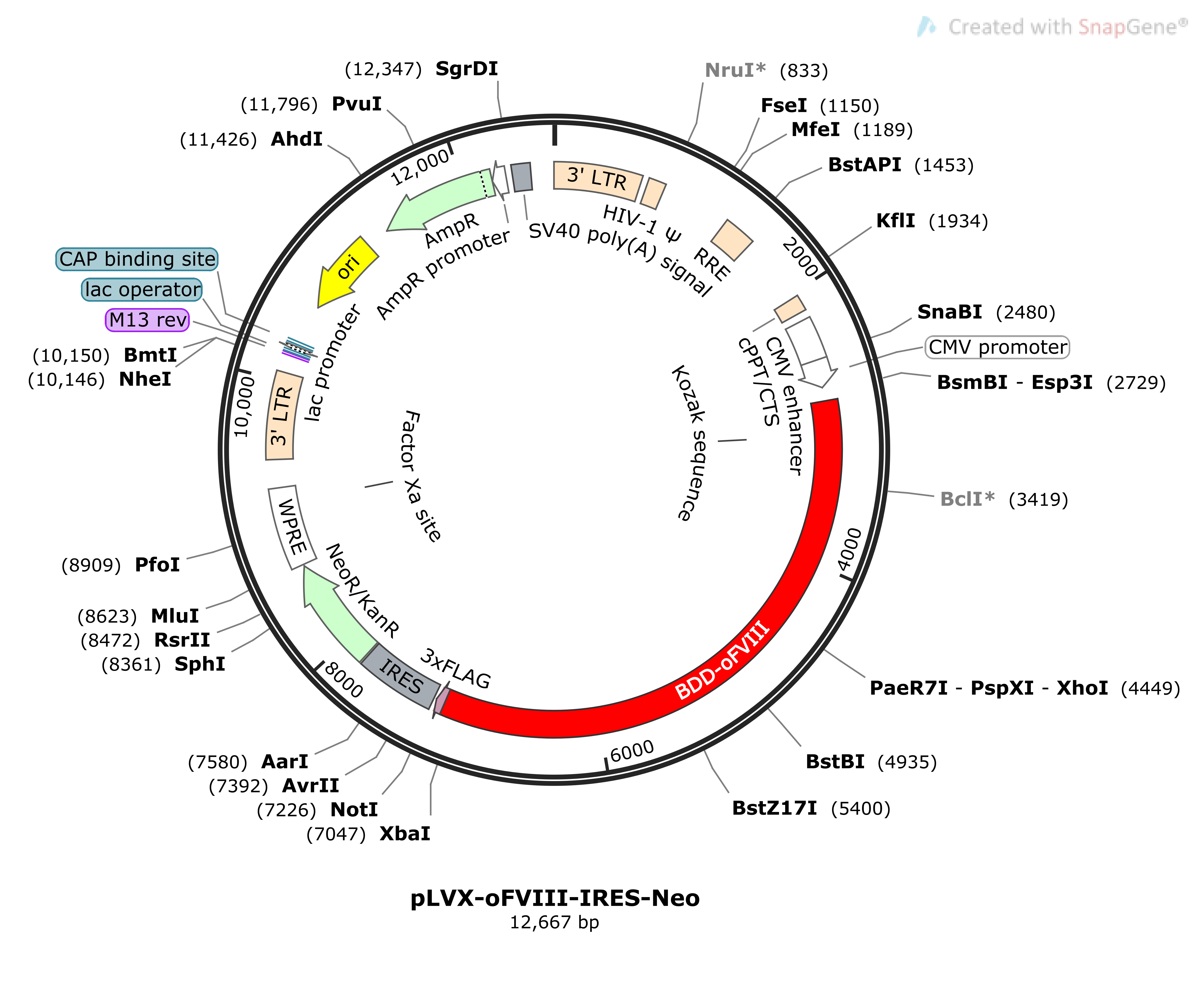

Supplement: Supplementary file 1 [file Image_1.tif]
